# MMI-0100 Ameliorates Dextran Sulfate Sodium-Induced Colitis in Mice through Targeting MK2 Pathway

**DOI:** 10.3390/molecules24152832

**Published:** 2019-08-03

**Authors:** Zhe Wang, Xue Ya Liang, Xin Chang, Yao Yan Nie, Chen Guo, Jin Hong Jiang, Min Chang

**Affiliations:** 1 School of Basic Medical Sciences, Xi’an Jiaotong University, Xi’an, Shanxi 710061, China; 2 Institute of Biochemistry and Molecular Biology, School of Life Sciences, Lanzhou University, Lanzhou 730000, China

**Keywords:** MMI-0100, dextran sodium sulfate (DSS), inflammatory bowel disease (IBD), MAPK-activated protein kinase II (MK2), apoptosis

## Abstract

**Backgrounds**: This study aimed to investigate the protective effects of MMI-0100, a cell-penetrating peptide inhibitor of MAPK-activated protein kinase II (MK2), on acute colitis induced by dextran sodium sulfate (DSS). Mice were injected intraperitoneally with different doses of MMI-0100 (0.5 and 1 mg/kg per day, six days). The physiological indexes, the parameters for colonic pathological injury and the intensity of inflammatory responses were evaluated by histological staining, quantitative PCR, western blotting, and immunostaining. MMI-0100 attenuated DSS-induced body weight loss, colon length shortening, and colonic pathological injury, including decreased myeloperoxidase (MPO) and inhibited inflammatory cell infiltration. MMI-0100 suppressed DSS-induced activation of CD11b^+^ and F4/80 positive cell, and dramatically decreased the expression of a series of pro-inflammatory cytokines such as TNF-α, IL-6, IL-1β, TGF- β, IFN-γ, IL-17A, COX-2 and iNOS. A TUNEL assay showed that MMI-0100 protected against DSS-induced apoptosis. This is consistent with the results of Western blotting assay in apoptosis-related proteins including Bcl-2, BAX, caspase-3. The anti-inflammatory effects of MMI-0100 on DSS-induced colitis were achieved by down-regulating the phosphorylation level of MK2, IκBα and p65 protein. The current study clearly demonstrates a protective role for MMI-0100 in experimental IBD.

## 1. Introduction

Inflammatory bowel disease (IBD) is a chronic and recurrent inflammatory disorder of the gastrointestinal (GI) tract which is characterized by excessively active immune responses and regular intestinal inflammation, and there is currently no curative treatment [1,2,3]. Targeting these immune mechanisms may lead to future preventive or therapeutic strategies for IBD [4]. Currently, immunosuppressive drugs including TNF-α antibody, methotrexate (MTX) and azathioprine (AZA) are the main therapeutic options for IBD [5,6,7]. However, due to their limitations in safety and efficacy, novel strategies with high safety and efficacy are urgently required.

The p38 mitogen-activated protein kinase (p38 MAPK) signaling pathways, a cascade contributing to inflammation, could play a key role in IBD pathophysiology [8,9]. Preclinical and clinical studies have evaluated the pharmacological effects of p38 MAPKα inhibitors such as AZD7624 (NCT01937338), VX-745 (NCT03435861), PH-797804 (NCT00559910) in inflammation diseases. Although targeting p38 MAPK is a candidate option for the treatment of inflammatory diseases, side effects of p38 inhibitors include cardiotoxicity, hepatotoxicity, and undisclosed CNS toxicity are unacceptable in safety profiles for drug development [10,11]. MAPK-activated protein kinase 2 (MK2), as the direct downstream substrate of p38 MAPK, play an essential role in the progression of inflammation [12,13,14]. Menon et al. demonstrated that MK2-dependent phosphorylation is a crucial checkpoint for cell fate in inflammation [12]. The suppression of MK2 activity also led to the downregulation of pro-inflammatory cytokine expression such as tumor necrosis factor-α (TNF-α), interleukin-1β (IL-1β), and IL-6 in bowel inflammation [15,16]. Therefore, MK2 is likely to be a promising option for the treatment of IBD. 

MMI-0100, a recently discovered cell-permeable peptide inhibitor of MK2, exhibits anti-inflammatory effects, including reduction of fibrosis, apoptosis and systolic dysfunction [17]. The sequence of MMI-0100, consists of 22 amino acids, is YARAAARQARAKALARQLGVAA. Phase 1a study investigated the effect of MMI-0100 on airway inflammation with inhaled lipopolysaccharide (LPS) (NCT02515396). Meanwhile, MMI-0100 is currently in clinical trials for the treatment of pulmonary fibrosis [18]. To the best of our knowledge, no data have been published to date regarding the role of MMI-0100 in experimental IBD. The aim of the present study was to determine whether MMI-0100 is able to attenuate dextran sodium sulfate (DSS) induced acute colitis and its underlying mechanism.

## 2. Results

### 2.1. MMI-0100 Ameliorates the Colon Damage and Colitis Induced by DSS in Mice

**Figure 1:**
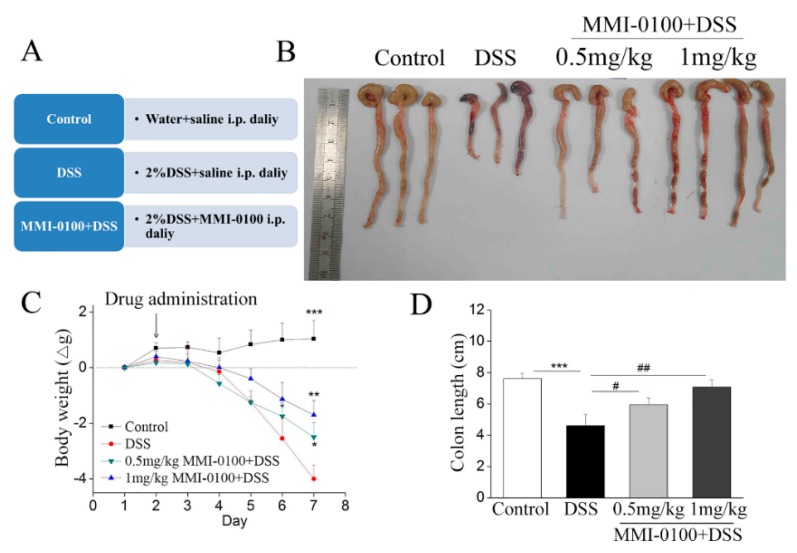
MMI-0100 alleviated the colon damage and colitis induced by DSS in mice. (**A**) Experimental design. Mice were given 2% DSS in drinking water for seven days. MMI-0100 was intraperitoneally (i.p.) given from day 2 to day 7. (**B**) Macroscopic appearances of colons from mice (**C**) Body weight changes of each group (*n* = 8–10 per group) after DSS induction of colitis. (**D**) The length of colons from each group of mice was measured. Data are presented as mean ± SD. In (**C**), * *p* < 0.05, ** *p* < 0.01 and *** *p* < 0.001 compared with DSS group; in (**D**), *** *p* < 0.001 for between control and DSS group, and ^#^ *p* < 0.05, ^##^ *p* < 0.01 for between MMI-0100 + DSS and DSS group.

As shown in Figure 1, DSS evoked severe inflammation in mice such as a dramatic loss of body weight, colon length shortening from days three to seven compared to control mice, which were consistent with previous publications [19,20]. However, intraperitoneal injection of MMI-0100 (0.5 and 1 mg/kg, i.p.) significantly ameliorated body weight loss (* *p* < 0.05 for 0.5 mg/kg MMI-0100 group vs. DSS group, ** *p* < 0.01 for 1 mg/kg MMI-0100 group vs. DSS group, *** *p* < 0.001 for control group vs. DSS group, Figure 1C), colon length shortening compared to the DSS group (^#^ *p* < 0.05 for 0.5 mg/kg MMI-0100 group vs. DSS group, ^##^ *p* < 0.01 for 1 mg/kg MMI-0100 group vs. DSS group, *** *p* < 0.001 for control group vs. DSS group, Figure 1D). 

**Figure 2:**
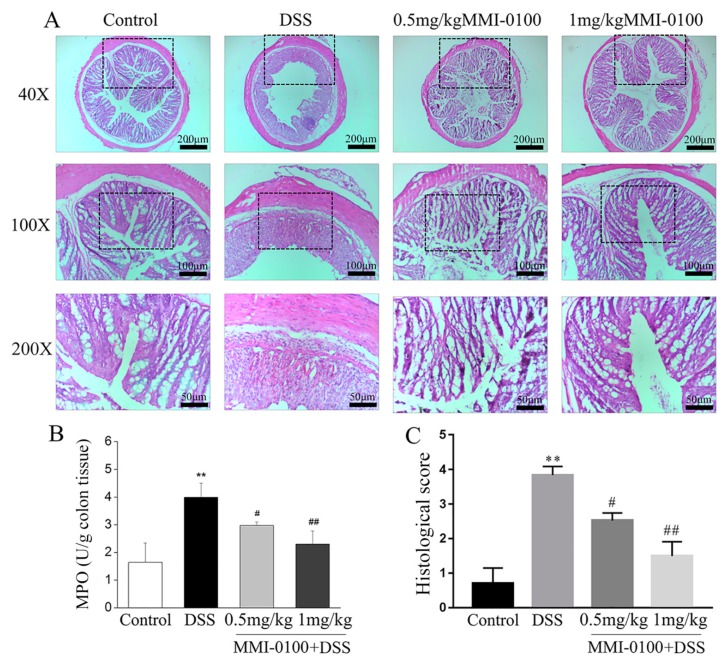
MMI-0100 prevented DSS-induced colon damage in mice. (**A**) Serial sections of colon tissues were stained with H&E staining. The scale bars represent 200 μm, 100 μm and 50 μm. (**B**) The MPO from each group of mice was measured. (**C**) The colonic sections of each animal were scored using a colitis score as described by method 5.7. Data are presented as mean ± SD. In (**B**) and (**C**), ** *p* < 0.01 for between control and DSS group, and ^#^ *p* < 0.05 and ^##^ *p* < 0.01 for between MMI-0100 + DSS and DSS group.

To further evaluate the severity of DSS-induced acute colitis, we investigated neutrophil infiltration in the colon tissues by using hematoxylin and eosin (H&E) staining. The experiments results showed that DSS-treated group evoked severe mucosal necrosis, accompanied by a large number of neutrophil infiltration as well as congestion and edema of the submucosa, whereas MMI-0100 dramatically relieved these symptoms (Figure 2A). Figure 2C exhibited histopathological scores of each group. Meanwhile, neutrophils are the effector cells of acute inflammation, play a role in the maintenance of intestinal homeostasis and pathogenesis of IBD. Myeloperoxidase (MPO) activities are often used as a marker of neutrophil infiltration in acute colitis. This is one of the main enzymes released upon neutrophil activation, and it is a heme protein that generates cytotoxic oxidants. In Figure 2B, the MPO activity in colons from MMI-0100-treated mice were significantly attenuated than that of the DSS group (** *p* < 0.01 for control group vs. DSS group, ^#^ *p* < 0.05 for 0.5 mg/kg MMI-0100 group vs. DSS group, ^##^ *p* < 0.01 for 1 mg/kg MMI-0100 group vs. DSS group, Figure 2B). 

### 2.2. MMI-0100 Reduces Pro-Inflammatory Cytokine Production and Inflammatory Cells Activation In Vivo

**Figure 3:**
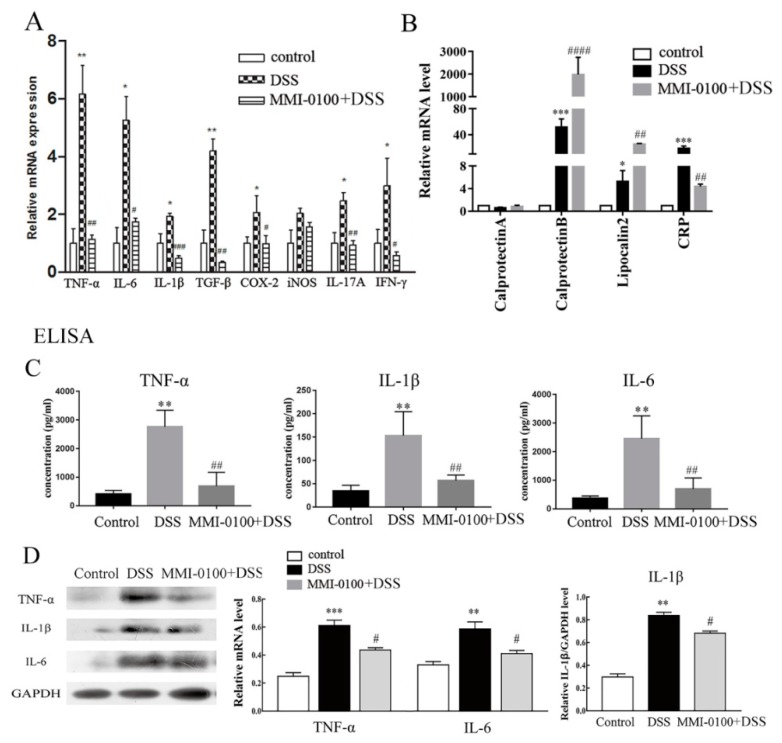
Effects of MMI-0100 on the production of inflammatory cytokines. (**A**) The mRNA expression level of inflammation-related cytokines TNF-α, IL-6, IL-1β, TGF- β, IFN-γ, IL-17A, COX-2 and iNOS in colon were determined by RT-qPCR. (**B**) The markers related to inflammation during acute colitis such as CRP, calprotectinA/B and lipocalin 2 were also measured by RT-qPCR. The production of inflammation-related cytokines TNF-α, IL-6 and IL-1β in colon were determined by ELISA (**C**) and WB (**D**). Data are presented as mean ± SD. * *p* < 0.05, ** *p* < 0.01 for between DSS group and control group; ^#^ *p* < 0.05, ^##^ *p* < 0.01 and ^###^ *p* < 0.001 for between MMI-0100 and DSS group.

To gain insight into the effect of MMI-0100 on the DSS-induced colitis, we assessed a series of pro-inflammatory cytokines at mRNA levels, such as TNF-α, IL-6, IL-1β, TGF-β, IFN-γ, IL-17A, COX-2 and iNOS. The results showed that the levels of these inflammatory mediators were dramatically increased in the colons of DSS-induced colitis model mice compared with control (*p* < 0.05). However, MMI-0100 (1 mg/kg, i.p.) significantly inhibited these pro-inflammatory factors (*p* < 0.05, Figure 3A). In addition, the markers related to inflammation or disease activity during acute colitis such as c-reactive protein (CRP), calprotectinA/B and lipocalin 2 were also measured. The results showed that MMI-0100 (1 mg/kg, i.p.) significantly increased the expression level of calprotectin B and lipocalin 2 (calprotectin B: *p* < 0.001 for control vs. DSS group, *p* < 0.0001 for MMI-0100 + DSS vs. DSS group; lipocalin 2: *p* < 0.05 for control vs. DSS group, *p* < 0.01 for MMI-0100 + DSS vs. DSS group, Figure 3B), and down-regulated in the increase of CRP induced by DSS (*p* < 0.001 for control vs. DSS group, *p* < 0.01 for MMI-0100 + DSS vs. DSS group, Figure 3B). As shown in Figure 3C,D, ELISA and WB experiments showed that the protein levels of TNF-α, IL-6 and IL-1β were also significantly increased after DSS treatment (ELISA: *p* < 0.01 for TNF-α, IL-6 and IL-1β, WB: *p* < 0.01 for IL-6 and IL-1β, *p* < 0.001 for TNF-α, Figure 3C,D). The administration of MMI-0100 (1 mg/kg, i.p.) to mice inhibited the production of these factors induced by DSS application (ELISA: *p* < 0.01 for TNF-α, IL-6 and IL-1β, WB: *p* < 0.05 for TNF-α, IL-6 and IL-1β, Figure 3C,D).

**Figure 4:**
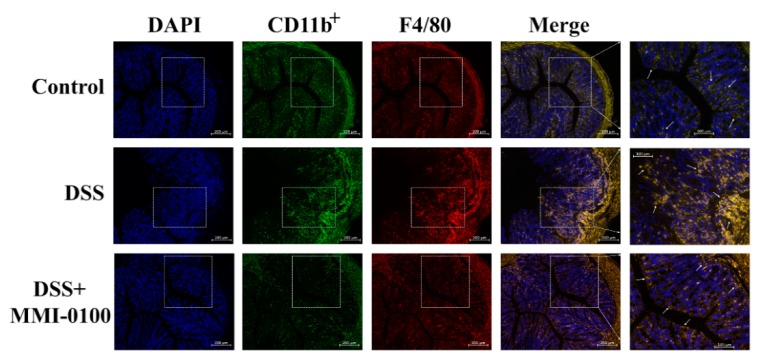
MMI-0100 attenuated DSS-induced macrophages activation. Representative images of CD11b^+^ (green) and F4/80 (red) cells in the colon of C57BL/6 mice. The scale bars represent 200 μm and 100 μm.

CD11b is expressed on the surface of many leukocytes, including neutrophils, monocytes, natural killer cells, macrophages and granulocytes, and F4/80 is a marker of macrophages [21]. Thus, we examined infiltration of inflammatory cells in colon tissues by using CD11b^+^ and F4/80 as a marker to monitor inflammation process. As shown in Figure 4, we observed more a great number of CD11b^+^ and F4/80 cells in the mucosa of the lesion site in DSS-treated mice. Conversely, fewer infiltrating cells were detected in the DSS + MMI-0100 group (Figure 4).

### 2.3. MMI-0100 Exerts Anti-Apoptosis Effects In Vivo

**Figure 5:**
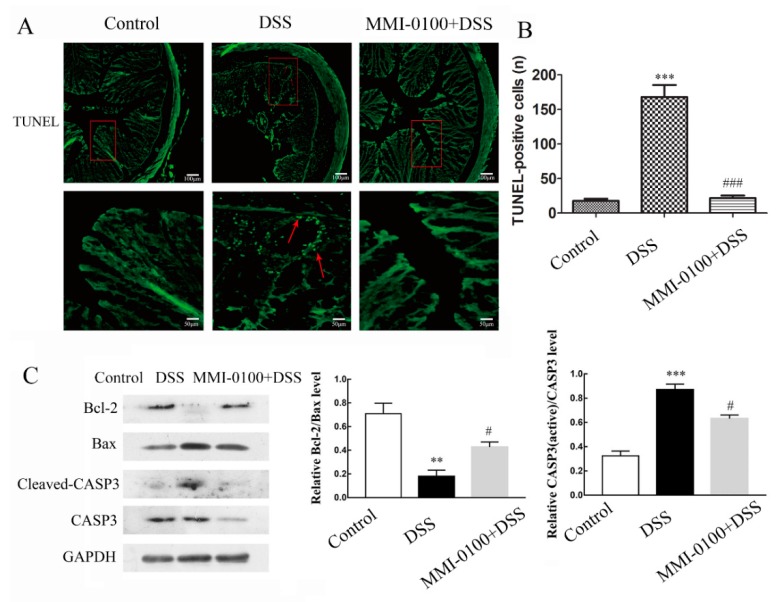
MMI-0100 exerted anti-apoptosis effects in DSS-induced colitis. (**A**) Sections of colon tissues were immunostained with TUNEL staining and observed by a ZEISS fluorescent microscope. The scale bars represent 100 μm and 50 μm. (**B**) The number of TUNEL-positive cells was quantitated as described by method 5.9. In (B), *** *p* < 0.001 for between control and DSS group, and ^###^ *p* < 0.001 for between MMI-0100 + DSS and DSS group. (**C**) The production of the apoptotic-related factor such as Bax, Bcl-2, caspase-3 and cleaved-caspase-3 were investigated by WB and quantified with statistical significances. Data are presented as mean ± SD. ** *p* < 0.01, *** *p* < 0.001 for between DSS group and control group; ^#^ *p* < 0.05 for between MMI-0100 and DSS group.

TUNEL assay showed that DSS treatment significantly enhanced the number of TUNEL-positive and apoptotic cell compared to the control (*p* < 0.001 for between control and DSS, Figure 5A,B). However, MMI-0100 application reduced the number of TUNEL-positive cells compared to the treated with DSS alone (*p* < 0.001 for between DSS and MMI-0100 + DSS, Figure 5A,B). Consistently, Western blotting analysis showed that MMI-0100 treatment remarkably up-regulated the expression of anti-apoptotic factor (Bcl-2, *p* < 0.05), and down-regulated the expression of pro-apoptotic factor (Bax andcaspase-3) (Figure 5B, *p* < 0.05 for both Bax and caspase-3). 

### 2.4. MMI-0100 Down-Regulates the Phosphorylation of MK2 and NF-κB Pathway in Mice

**Figure 6:**
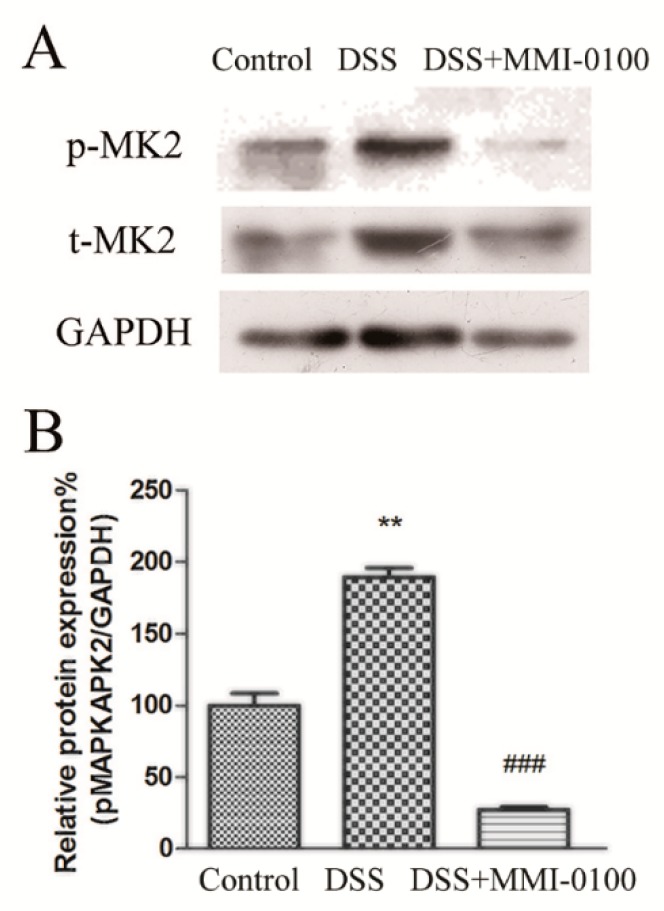
MMI-0100 prevented DSS-induced colon colitis by activating MK2 signaling pathway. (**A**,**B**) The production of p-MK2, MK2 was investigated by WB and quantified with statistical significances. Data are presented as mean ± SD. ** *p* < 0.01 for between DSS group and control group; ^###^ *p* < 0.001 for between MMI-0100 and DSS group.

**Figure 7:**
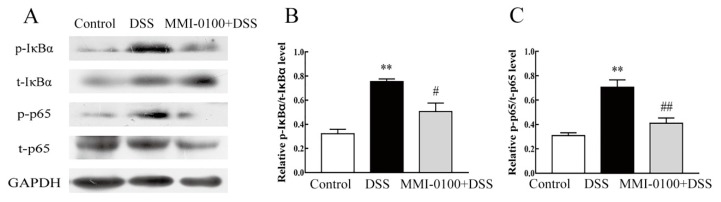
MMI-0100 prevented DSS-induced colon colitis by activating NF-κB signaling pathway. (**A**–**C**) The production of p-IκBα, IκBα, p-p65 and p65 were investigated by WB and quantified with statistical significances. Data are presented as mean ± SD. ** *p* < 0.01 for between DSS group and control group; ^#^ *p* < 0.05 and ^##^ *p* < 0.01 for between MMI-0100 and DSS group.

MMI-0100 is a cell-penetrating peptide inhibitor of MK2. Gorska et al. demonstrated that MK2 and its substrate HSP27 are essential for sustained NF-κB activation [22,23]. Therefore, to gain insight into the mechanism of MMI-0100 in the suppression of colitis, we further investigated the expression of MK2 and NF-κB signaling pathways by Western blotting. As shown in Figure 6 and Figure 7, the phosphorylation levels of MK2, IκBα and p65 were significantly up-regulated in the colon of DSS-induced colitis model mice (*p* < 0.01 for pMK2/tMK2, *p* < 0.01 for both IκBα and p65), while MMI-0100 treatment remarkably decreased the level of p-MK2, p-IκBα and p-p65 (*p* < 0.001 for pMK2/tMK2, *p* < 0.05 for IκBα and *p* < 0.01 for p65). 

## 3. Discussion

In general, DSS-induced colitis is established to evaluate the model of human inflammatory bowel disease [24,25,26]. In this study, MMI-0100 attenuated DSS-induced inflammatory signs such as body weight loss, colon length shortening and colonic tissue damage. MPO activities, as a marker of infammatory cells infiltration, were increased by DSS. However, it was decreased in the colon of DSS-induced colitis mice model after MMI-0100 application. DAI scores of MMI-0100 group were lower than the DSS-treated group as well. Moreover, MMI-0100 suppressed activation of CD11b+ and F4/80 positive cell induced by DSS, and dramatically decreased the expression level of a series of pro-inflammatory cytokines such as TNF-α, IL-6, IL-1β, TGF- β, IFN-γ, IL-17A, COX-2 and iNOS. Previous articles have reported protective roles of calprotectin and lipocalin 2 against colitis [27,28]. Our data indicated that MMI-0100 significantly increased the expression level of calprotectin B and lipocalin 2, and down-regulated in the increase of CRP induced by DSS. These data suggested that MMI-0100 successfully reversed DSS-induced acute colitis by inhibiting the production of a series of pro-inflammatory cytokines in colons and exerted anti-inflammatory effects against inflammatory disease. Our findings were supported by the previous study that MK2 peptide inhibitors showed a decrease in TNF-α and IL-6, suggesting that inhibited MK2 activity could be considered as a potential therapy for applications involving inflammation [12,29,30]. 

Accordingly, we inferred that MMI-0100 may protect against the colon damage and apoptosis induced by the inflammatory response. A TUNEL assay showed that more apoptotic cells were detected in DSS induced colitis group compared to control or MMI-0100 group. Consistent with TUNEL assay, western blotting experiments showed that MMI-0100 increased the expression of anti-apoptotic factor (Bcl-2) and decreased the level of pro-apoptotic factor (Bax), suppressed the expression of cleaved Caspase-3, and finally, protected intestinal epithelial cells from apoptosis. These results suggest that MMI-0100 could protect intestinal epithelial cells against the DSS-induced toxic damage, which is consistent with previous reports that MMI-0100 has protective effects under many unfavorable conditions [31,32].

In the past few years, MK2 was activated by a wide range of inflammatory conditions such as LPS, Aβ, and its catalytic activity was required for inflammatory cells infiltration and cytokine production. Disruption of MK2 leads to a reduction in pro-inflammatory cytokines production, which has been demonstrated in several inflammatory diseases [29,30,33]. Thus, MK2 is a potential drug target for inflammatory diseases. The underlying mechanism of were not clear. We unraveled the underlying mechanisms whether these anti-inflammatory effects of MMI-0100 in DSS-induced acute colitis may be involved in MK2 system. The current study clearly demonstrates that the phosphorylation of MK2 was significantly up-regulated in the colon of DSS-induced colitis model mice while MMI-0100 treatment remarkably decreased the level of the phosphorylation of MK2 in colon. 

Given that MK2 is essential for biosynthesis of TNF and other cytokines at post-transcriptional level, mainly regulating by phosphorylating translation-inhibiting protein tristetraprolin (TTP) [34,35,36,37]. In addition, Gorska et al. demonstrated that MK2 and its substrate HSP27 are essential for sustained NF-κB activation [22,23]. Therefore, to gain insight into the mechanism of MMI-0100 in the suppression of colitis, we evaluated the underlying mechanism of the anti-inflammatory effects of MMI-0100 on the activation of NF-κB signaling pathway molecules such as IκBα and p65 in colon. Strikingly, our results revealed that MMI-0100 treatment pronouncedly suppressed the DSS-induced increase of phosphorylation of IκBα and p65 by western blotting. MMI-0100 is a peptide inhibitor of MK2. Some articles reported that MK2 prevents premature export of NF-κB from the nucleus in inflammatory diseases [22,23], suggesting that NF-κB signaling suppression may also contribute to the protective role for MMI-0100 in experimental IBD. All data showed that MMI-0100 might exert anti-inflammatory activities through the regulation of MK2 signaling proteins and the NF-κB signaling pathway in DSS-induced acute colitis. Meanwhile, these data suggest that MMI-0100/MK2 system may provide a new potential target for treatment of IBD.

## 4. Materials and Methods

### 4.1. Animals

Male C57BL/6 mice (weighing 20–22 g, 8–10 weeks-old) were purchased from the Experimental Animal Center of Lanzhou University (Lanzhou, China). All mice were housed in cages (five animals/cage) with free access to tap water and food in an animal room, which were maintained at 22 ± 2 °C in a 12-h light (8:00)/dark (20:00) cycle. All animal protocols in this study were performed under the guidelines of the Ethics Committee of Lanzhou University, China (permit number: SYXK Gan 2009–0005).

### 4.2. Drugs

MMI-0100 (YARAAARQARAKALARQLGVAA) was synthesized by a standard Fmoc-based solid-phase synthetic method, and detailed methods refer to our previous reports [38]. Dextran sulfate sodium (DSS, MW: 36–50 kDa) was purchased from MP biomedicals inc (Irvine, CA, USA). Myeloperoxidase (MPO) activity assay kit was purchased from Nanjing jiancheng bioengineering institute (Nanjing, China). Hematoxylin and eosin staining kit was purchased from beyotime biotechnology (cat number: C0105, Shanghai, China). MMI-0100 was dissolved in saline to 1 mg/kg/mL, stored at −20 °C after being packed, and was diluted in saline immediately before injected (1 mg/kg/10 mL). DSS was dissolved in drinking water.

### 4.3. Establishment Mouse Acute Colitis Model Induceed by DSS

Acute colitis was induced by DSS in drinking water. Male mice received either drinking water or 2% (*w*/*v*) DSS drinking water for seven days [39]. A total of 42 mice were randomly assigned to four groups as follows: i) Control group, ii) DSS-treated group, iii) DSS + MMI-0100 (0.5 mg/kg, i.p.)-treated group and iv) DSS + MMI-0100 (1 mg/kg, i.p.)-treated group. MMI-0100 was given intraperitoneally from day two to day seven.

### 4.4. Reverse Transcription-Quantitative Polymerase Chain Reaction (RT-qPCR) Analysis

The colons were removed from mice. RT-qPCR was performed according to the previous reports and manufacturer’s instructions [40]. Total RNA was extracted using Trizol reagent (TaKaRa, Dalian,China) and 1 µg of RNA sample was reverse transcribed into complementary DNA (cDNA) with the 5X PrimeScript RT Master Mix (TaKaRa). Amplification was carried out in a 25 µL the reaction mixture, and was run under the following conditions: 95 °C for 30 s, followed by 40 cycles of 95 °C for 5 s, 58 °C for 30 s and 72 °C for 30 s. The primers in RT-qPCR assay were shown in Table 1 and were used to identify the expression level of IL-6, TNF-α, IL-1β, TGF- β, IFN-γ, IL-17A, COX-2, iNOS, s100a8 (calprotectin A), s100a9 (calprotectin B), Lipocalin2 and CRP genes in control, DSS-treated, MMI-0100 (0.5 and 1 mg/kg) + DSS-treated groups. GAPDH was used as the internal control.

### 4.5. Western Blotting

Western blotting was carried out following manufacturer’s instructions. Briefly, total proteins were extracted with RIPA buffer containing protease inhibitor (Gibco, Shanghai, China). The protein samples (40 µg of each lane) isolated from colons were separated by SDS-PAGE and then electrophoretically transferred onto polyvinylidene fluoride (PVDF) membranes. Membranes were blocked with 5% fat-free milk in PBS for 2 h and incubated overnight at 4 °C with specific first antibodies. GAPDH was used as a loading control. The first antibodies in WB assay were shown in Table 2. Subsequently, after being washed threetimes with PBS, the membranes were incubated with horseradish peroxidase-conjugated secondary antibodies (cat number: A0208, 1:1000, Beyotime, Shanghai, China). The reaction was visualized with a chemiluminescence reagent provided with an ECL kit (Thermo, Fisher Scientific, Inc., Shanghai, China) and exposed to the film. The intensity of the blots was quantified by densitometry.

### 4.6. Enzyme-Linked Immunoassay (ELISA)

Total proteins from the colons of mice in each group were extracted with RIPA lysis buffer containing protease inhibitor (Gibco, Thermo Fisher Scientific, Inc., Shanghai, China). Total proteins were determined using the BCA^TM^ protein assay kit (Sangon Biotech, Shanghai, China). IL-1β, IL-6 and TNF-α in colon tissue were measured by ELISA kits (cat number: E-EL-M0049 for TNF-α, cat number: E-EL-M0044 for IL-6, cat number: E-EL-M0037 forIL-1β, Elabscience, Wuhan, China ), according to the manufacturer’s instructions.

### 4.7. Histological Analysis

A histopathological assay was performed according to previous report [41]. Briefly, mice were sacrificed, and colons were removed. Whole colon tissues were fixed in 4% paraformaldehyde at 4 °C overnight. The 8-μm sections were stained with H&E staining kit (cat number: C0105, Beyotime, Shanghai, China) using standard methods. The images were performed by an ordinary optical microscope (Olympus, Tokyo, Japan). According to the methods in the literatures [42,43,44], histopathological scores were assigned for 4 grades with the following criteria: the destruction of the crypt structure (1: none, 2: basal 1/3, 3: basal 2/3, 4: only surface epithelium intact), the degree of inflammatory cell infiltration (1: none, 2: slight, 3: moderate, and 4: severe), and the depth of the lesions (1: none, 2: mucosal, 3: mucosal and submucosal, and 4: transmural).

### 4.8. Assessment of Myeloperoxidase (MPO) Activity

Neutrophil infiltration into inflamed colonic mucosa was quantified by assessment of MPO activity using the O-dianisidine method. Protein extracts from colonic tissue were used to assess MPO levels according to the manufacturer’s instructions.

### 4.9. Immunostaining and TUNEL Staining

Mice were sacrificed and colons were removed. The colon tissues were cut into consecutive frozen sections and then incubated overnight with CD11b+ or F4/80 antibody. The secondary antibody (1:200, Beyotime, Shanghai, China) was applied to the sections for 40 min at 37 °C. Then the slides were counter-stained with DAPI for 30 min, and were washed in water for 5 min. The images were performed by a fluorescent microscope (ZEISS International, optical and optoelectronic technology, San Diego, CA, USA).

For TUNEL assay, the colon tissues were cut into consecutive frozen sections and then incubated with TUNEL dye liquor (cat number: C1088, Beyotime, China) for 30 min, and were washed in water for 5 min. The localized green fluorescence of the apoptotic cells was detected by a fluorescent microscope (ZEISS International, optical and optoelectronic technology, USA). Five randomly selected fields (magnification × 200) were counted in each colonic section from10 section. The average of TUNEL-positive cells in 10 sections was used to present the apoptotic index.

### 4.10. Statistical Analysis

All data were presented as mean ± SEM. The statistical analysis was conducted by one-way ANOVA followed by Bonferroni’s post-hoc test by using SPSS 19.0. *p* < 0.05 was considered statistically significant.

## 5. Conclusions

Overall, the present study firstly demonstrated that MMI-0100 relieved DSS-induced acute colitis and provided protective role against DSS-induced inflammatory response and apoptosis in vivo. The underlying mechanisms may be related with down-regulation of the phosphorylation of MK2 and NF-κB signaling pathway inactivation by MMI-0100 treatment. Taken together, the current study clearly demonstrates that MMI-0100 plays an important protective role in experimental IBD model mice, suggesting that the MMI-0100/MK2 system may provide a new potential target for the treatment of IBD.

## Figures and Tables

**Table 1:** Primers used in the reverse transcription-quantitative PCR in the present study.

Gene	Prime	Sequence (5′–3′)
IL-1β	sense	CAGCTTCAAATCTCGCAGCA
	anti-sense	CTCATGTCCTCATCCTGGAAGG
TNF-α	sense	ACTCCCAGGTTCTCTTCAAGG
	anti-sense	GGCAGAGAGGAGGTTGACTTTC
iNOS	sense	CGCAGCTGGGCTGTACAAAC
	anti-sense	CTGTGGCTCCCATGTTGCATT
IL-6	sense	ACAACCACGGCCTTCCCTA
	anti-sense	TCATTTCCACGATTTCCCAGA
TGF-β	sense	TTCAGCCACTGCCGTACAACTC
	anti-sense	AGCAACAATTCCTGGCGTTACCT
IFN-γ	sense	CAAGTTTGAGGTCAACAACCCAC
	anti-sense	GACTCCTTTTCCGCTTCCTGA
IL-17A	sense	CACCCTGGACTCTCCACCG
s100a8 (calprotectin A) s100a9 (calprotectin B) Lipocalin2 CRP	anti-sense sense anti-sense sense anti-sense sense anti-sense sense anti-sense	GCTTTCCCTCCGCATTGACA ATCTTTCGTGACAATGCCGTCT GCCACACCCACTTTTATCACCA GCAGCATAACCACCATCATCGAC AGATCAACTTTGCCATCAGCATC AAACAGAAGGCAGCTTTACGAT GCCACTTGCACATTGTAGCTCT TTCCCAAGGAGTCAGATACTTCC TCAGAGCAGTGTAGAAATGGAGA
GAPDH	sense	GCCACAGACGTCACTTTCCTAC
	anti-sense	CGGGAACACAGTCACATACCA

IL-1β, interleukin-1β; TNF-α, tumor necrosis factor-α; iNOS, inducible nitric oxide synthase; IL-6, interleukin-6; TGF-β, transforming growth factor-β; IFN-γ, Interferon gamma; IL-17A, interleukin-17A; s100a8, calprotectin A; s100a9, calprotectin B; CRP, c-reactive protein.

**Table 2:** Antibody information used in this experiment.

Antibody Name	Dilution Concentration	Cat Number	Company
Anti-F4/80 Rabbit pAb	1:100	GB11027	Servicebio
Anti-CD11b^+^ Rabbit pAb Anti-TNF-α Rabbit pAb Anti-IL-1β Rabbit mAb Anti-IL-6 Rabbit mAb	1:100 1:1000 1:500 1:500	GB11058 3707S sc-52012 sc-57315	Servicebio CST, USA Santa Cruz, USA Santa Cruz, USA
Anti-p-MK2 Rabbit mAb	1:1000	3007S	CST, USA
Anti-t-MK2 Rabbit mAb	1:500	D16188-0025	BBI Life Sciences
Anti-Bcl-2 Rabbit mAb	1:500	sc-7382	Santa Cruz, USA
Anti-Bax Rabbit pAb	1:500	D120073-0025	BBI Life Sciences
Anti-c-Caspase-3Rabbit mAb	1:500	AC033	Beyotime
Anti-Caspase-3 Rabbit pAb	1:500	AC030	Beyotime
Anti-pIκBα Rabbit mAb	1:500	sc-8404	Santa Cruz, USA
Anti-IκBα Rabbit mAb Anti-p-p65 Rabbit mAb Anti-p65 Rabbit mAb	1:500 1:1000 1:500	sc-1643 3033S sc-8008	Santa Cruz, USA CST, USA Santa Cruz, USA
GAPDH Rabbit mAb	1:1000	5174S	CST, USA
Second antibody	1:1000	A0208	Beyotime
